# Preliminary Flu Outbreak Prediction Using Twitter Posts Classification and Linear Regression With Historical Centers for Disease Control and Prevention Reports: Prediction Framework Study

**DOI:** 10.2196/12383

**Published:** 2019-06-23

**Authors:** Ali Alessa, Miad Faezipour

**Affiliations:** 1 Department of Computer Science and Engineering University of Bridgeport Bridgeport, CT United States; 2 Institute of Public Administration Riyadh Saudi Arabia; 3 Department of Biomedical Engineering University of Bridgeport Bridgeport, CT United States

**Keywords:** FastText, influenza, machine learning, social networking site, text classification

## Abstract

**Background:**

Social networking sites (SNSs) such as Twitter are widely used by diverse demographic populations. The amount of data within SNSs has created an efficient resource for real-time analysis. Thus, data from SNSs can be used effectively to track disease outbreaks and provide necessary warnings. Current SNS-based flu detection and prediction frameworks apply conventional machine learning approaches that require lengthy training and testing, which is not the optimal solution for new outbreaks with new signs and symptoms.

**Objective:**

The objective of this study was to propose an efficient and accurate framework that uses data from SNSs to track disease outbreaks and provide early warnings, even for newest outbreaks, accurately.

**Methods:**

We presented a framework of outbreak prediction that included 3 main modules: text classification, mapping, and linear regression for weekly flu rate predictions. The text classification module used the features of sentiment analysis and predefined keyword occurrences. Various classifiers, including FastText (FT) and 6 conventional machine learning algorithms, were evaluated to identify the most efficient and accurate one for the proposed framework. The text classifiers were trained and tested using a prelabeled dataset of flu-related and unrelated Twitter postings. The selected text classifier was then used to classify over 8,400,000 tweet documents. The flu-related documents were then mapped on a weekly basis using a mapping module. Finally, the mapped results were passed together with historical Centers for Disease Control and Prevention (CDC) data to a linear regression module for weekly flu rate predictions.

**Results:**

The evaluation of flu tweet classification showed that FT, together with the extracted features, achieved accurate results with an *F*-measure value of 89.9% in addition to its efficiency. Therefore, FT was chosen to be the classification module to work together with the other modules in the proposed framework, including a regression-based estimator, for flu trend predictions. The estimator was evaluated using several regression models. Regression results show that the linear regression–based estimator achieved the highest accuracy results using the measure of Pearson correlation. Thus, the linear regression model was used for the module of weekly flu rate estimation. The prediction results were compared with the available recent data from CDC as the ground truth and showed a strong correlation of 96.29% *.*

**Conclusions:**

The results demonstrated the efficiency and the accuracy of the proposed framework that can be used even for new outbreaks with new signs and symptoms. The classification results demonstrated that the FT-based framework improves the accuracy and the efficiency of flu disease surveillance systems that use unstructured data such as data from SNSs.

## Introduction

### Background

According to the Centers for Disease Control and Prevention (CDC), flu is a serious contagious respiratory illness that can lead to hospitalization and sometimes death. About 250,000 to 500,000 deaths occur worldwide each year because of flu. Flu is common during some seasons, but there can be deadly outbreaks that spread suddenly in a community.

Social networking sites (SNSs) are tools that include big data about users and their shared thoughts and ideas, in addition to real-time data of users’ conversations and statuses. The amount of data, aside from the growth of SNS users, represents the important role of SNSs in real-time analysis and predictions in many areas, including the area of public health [1]. SNSs provide an efficient resource to conduct disease surveillance and a communication tool to prevent disease outbreaks [2].

To produce outbreak reports, typical disease surveillance systems depend on official statistics based on patient visits [3]. In the United States, these reports are produced by the CDC to inform health care providers about certain disease outbreaks such as influenza outbreaks. CDC publishes flu-related reports using the US Influenza Like Illness Surveillance Network (ILINet) that gathers flu-related information of outpatients from hundreds of health care providers around the United States. ILINet shows accurate results in detecting flu outbreaks, but it is costly and takes a long time to issue the required reports. It is crucial for any disease surveillance system to collect related data and provide the reports as early as possible to prevent the spread of the disease. To this end, many solutions have been proposed to generate earlier outbreak warnings. Examples include volumes of telephone calls, over-the-counter drug sales [3], search engine logs [4-9], and data from SNSs that can be used for real-time analysis for better services [10-14]. Analysis of search engine logs, such as Google Flu Trend (GFT), estimates the percentage of ILI cases using flu correlated queries. In 2013, GFT overpredicted the percentage of the ILI cases by the double [15]. Compared with different resources used for surveillance, that is, search engine logs, data from SNSs are more descriptive and available to the public. Because SNSs provide certain information about users, the collected data can be used to simulate the spread of disease outbreaks in connected geographic areas with temporal analysis [15].

In this study, we relied on the Twitter microblog to conduct minute-by-minute analysis to track the high frequency of posted messages. We present a framework to track influenza trends through Twitter postings. The framework includes preprocessing, feature extraction, Twitter documents classification, documents weekly-mapping, and weekly flu rate predictions. The preprocessing phase includes stemming and removal of stop words and ineffective characters, which are nonalphanumeric tokens. Thereafter, the preprocessed data are used to extract features to be passed to a tweet classifier to distinguish between flu-related tweets and unrelated ones. The flu-related documents are then mapped on a weekly basis. Finally, the mapped results are passed together with historical CDC data to an estimator for flu trend predictions.

The data generated from SNSs are valuable for real-time analysis and outbreak predictions, but its volume is huge. Therefore, one of the main challenges in analyzing this huge volume of data is to find the best approach for accurate analysis in a time-efficient manner. Current Twitter-based flu detection and prediction frameworks apply conventional machine learning approaches that require lengthy training and testing, which is not the optimal solution to be used for a new outbreak with new signs and symptoms. Regardless of the analysis time, many studies only report the accuracy of different machine learning approaches. Thus, more efficient solutions are required for accurate results with less processing time. In this study, we demonstrate that using FastText (FT) can enhance the efficiency of Twitter-based flu outbreak prediction models. Originally, FT became an efficient text classifier that was proposed by Facebook. FT performs more quickly than deep learning classifiers for training and testing procedures and produces comparably accurate results. The FT classifier can train more than a billion words in about 10 min and then predict multiple classes within half a million sentences in less than a minute [16].

The aim of this study was to develop an efficient Twitter-based model that provides accurate results with less processing time to predict seasonal and serious outbreaks such as H5N1. This study presents an accurate and efficient FT-based framework to generate influenza trend predictions from Twitter. In addition to the typical textual features, the proposed framework uses the features of text sentiment analysis and the occurrences of predefined topic keywords to distinguish between flu-related tweets and unrelated ones to be passed together with historical CDC data to an estimator module for weekly flu rate predictions. The main contributions of this study can be summarized as follows: (1) demonstrating that FT classifier can improve the efficiency of tweet classification; (2) including sentiment analysis of the analyzed posts as a feature to improve the accuracy of the classification results; (3) examining various conventional machine learning algorithms for flu-related tweets; (4) proposing a weekly flu rate estimator based on the linear regression model that uses a combination of the classification results and historical CDC data; and (5) examining, in addition to the linear regression model, several regression techniques for weekly flu rate estimation.

### Problem Definition

SNS postings can be seen as triggers for different event predictions such as disease outbreaks. Discovering knowledge from the posts for flu surveillance models requires an efficient approach of text processing. It includes gathering the related text (posts) about the disease and then issuing necessary reports at an early stage, which is crucial for outbreak prevention. Because the gathered data is unstructured, the first step is to preprocess the unstructured content to analyze the data and produce the results in an understandable way. The second step is feature extraction, which is key to performance enhancement. The third step is knowledge extraction using machine learning techniques for text classification, which includes model training and testing. A post on a microblogging site is then classified into either related or unrelated classes, as can be seen from the following example:

Related: *I’m sick, I got flu yesterday.*

Unrelated: *I’m sick of school.*

Our literature survey indicates that most of the existing frameworks use conventional machine learning classifiers [17]. These approaches require a longer time duration for the training process. A new outbreak may require retraining the used prediction model with its new signs and symptoms to consider the related posts. Thus, such approaches are not optimal solutions for new deadly flu outbreaks.

The proposed framework using FT classifier, together with the extracted features, which have not been previously used for Twitter-based flu surveillance models, aims to extract related posts faster with a comparable accuracy. Thus, it can be used for urgent cases to stop the spread of a new deadly outbreak. Improving the efficiency, along with the accuracy of text classification is important for text-based surveillance systems for generating early reports. To develop a flu outbreak prediction framework, the classified tweets are then passed together with historical CDC data to an estimator module for weekly flu rate predictions.

### Previous Work

Previous works about Twitter-based flu surveillance systems include machine learning methods to filter unrelated flu posts. A selected classifier is trained with an annotated dataset using a set of features. The literature discusses various detection and prediction models that used different classification methods with different feature extraction techniques.

Broniatowski et al [18] and Lamb et al [19] proposed a multilevel classification model that included a binary classifier to distinguish between flu-related and unrelated flu tweets. The preclassifiers were used to filter unwanted posts such as health-irrelevant posts to increase the efficiency of the flu-related/unrelated classifier in further stages/levels. Here, the researchers demonstrated that multilevel classification can improve classification accuracy.

Aramaki et al [20] proposed a framework that consisted of 2 parts: a tweet crawler and a support vector machine (SVM)–based classifier that was used to extract only the actual influenza tweets and excluded the unrelated ones such as news and questions. The initial dataset for their study was collected between November 2008 and June 2010. It included 300 million general tweets. The dataset was then filtered using the “Influenza” keyword to get a set of only flu-related tweets that contained 400,000 tweets. The flu-related dataset was divided into 2 parts: a training dataset that contained 5000 tweets (November 2008) and a test dataset that contained all the remaining tweets between December 2008 and June 2010. The training dataset was assigned to a human annotator to label each tweet as either positive or negative. A tweet was labeled positive if it met 2 conditions. First, the flu tweet should be about the person who posted the tweet or about another person in a nearby area (maximum an area of the city). If the distance is unknown, the tweet is considered negative. Second, the flu tweet should be an affirmative sentence and in present tense or past tense with maximum period of 24 hours, which can be checked using specific keywords such as ”yesterday.” The SVM classifier was implemented using the bag-of-words (BoW) feature representation. The authors compared the accuracy of the SVM-based classifier with *6* other different machine learning methods and found that SVM was the most accurate method.

Santos et al [21] also applied SVM-based classification to detect FLI in Portugal using twitter posts. For the purpose of training and testing, a dataset with 2704 posts was manually annotated with 650 textual features. A subset of the annotated dataset was used to train the classifier. The classified tweets, together with search queries, were applied to a regression model as predictors. The classifier was implemented using the BoW feature representation, and the feature selection process was based on a mutual information (MI) value that was used to pick the best set of features. In this approach, each feature is applied to a true class, and then the MI value is assigned to the feature. The value of MI is based on how the feature is related to the true class. A feature with high MI value represents being more related to the true class.

Yang et al [22] proposed the first SVM-based method to predict flu trends from Chinese microblogging sites in Beijing. The collected data for their study included 3,505,110 posts between September 2013 and December 2013. Among those, 5000 random posts were selected for manual annotation (sick and not sick labels) to be used for training and testing purposes. Of these, 285 of the sick posts and 285 of the not sick posts were picked for training. For higher accuracy, word-based features were used instead of character-based features. In addition, the term frequency-inverse document frequency (TF-IDF) method was considered for weighting. Different classifiers were compared to decide which classifier would be best for the problem. The authors found that SVM was the best classifier for big data problems.

Byrd et al [23] proposed a framework based on the Naïve Bayes classifier. The framework consisted of preprocessing and flu tweet classification based on sentiment analysis. Three machine learning algorithms were evaluated. The results indicated that the highest accuracy algorithm was the Naïve Bayes classifier. The classifier was implemented using the Stanford CoreNLP (a natural language processing [NLP] software) and trained using the OpenNLP training dataset, which included 100 annotated tweets. Sentiment analysis is considered accurate when there is a match between the predicted sentiment polarity and the manual assigned opinion of the sentiment. The researchers found that Naïve Bayes was the most accurate classifier with a rate of 70% match.

## Methods

### Proposed Framework

The proposed framework, which includes a classification model for flu posts, published on the Twitter microblogging site, is implemented using the Cross Industry Standard Process for Data Mining (CRISP–DM). It is a well-known standard for implementing data mining frameworks. This standard includes the following 6 steps [24]:

- Business understanding
- Data understanding
- Data preparation
- Modeling
- Evaluation
- Deployment

**Figure 1:**
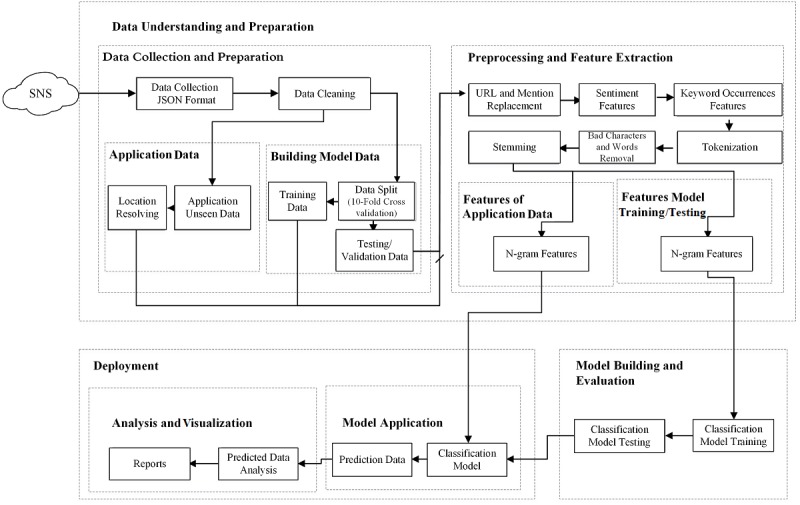
Methodology for text classification of flu tweets. SNS: social networking sites; JSON: JavaScript Object Notation.

On the basis of the CRISP–DM standard, the methodology for this study is presented in Figure 1.

### Data Collection and Preparation

#### Classification Model Data

For classification model training and testing, we prepared a labeled dataset that is a combination of multiple manually labeled datasets obtained from [19,25]. This makes the total instances of the merged dataset 10,592 tweets (5249 flu-related and 5343 flu-unrelated posts). Due to Twitter guidelines, the tweets in the obtained datasets were released with tweet IDs instead of the text of the tweets. Therefore, we developed a script that works together with the Twitter application programming interface (API) to retrieve the corresponding tweet texts using the given IDs. The collected tweets were cleaned to include only the texts for training and testing purposes. Then, we divided the merged dataset into 2 parts: training set and testing set.

##### Twitter Influenza Surveillance Dataset

The labeled dataset obtained from Lamb et al [19] was initially filtered to contain any posts that have flu-related keywords. Then, every post in the dataset was labeled manually. It was prepared to train and test 3 flu-related classifiers that were used as a part of an algorithm for seasonal flu predictions. The dataset was divided into 3 sets, 1 for each classifier. The first set consisted of tweets that were labeled as either flu-related tweets or unrelated. The second one had tweets with labels of flu infections or flu awareness. The tweets in the last set were labeled as either the flu tweet being about the author or about someone else. For our training dataset, we considered the tweets in the second and third datasets as flu-related tweets and combined all of them with only 2 labels: flu-related or unrelated.

##### Sanders Dataset

The labeled dataset obtained from Sanders [25] was prepared manually to train and test sentiment analysis algorithms. Each record in the dataset was annotated with a sentiment label, indicating a feeling toward either Google, Twitter, Microsoft or Apple. The labels were as follows: positive, neutral, negative, and irrelevant. Because this dataset was prepared for sentiment analysis of topics that are not related to flu, we used all the tweets in this dataset, with the exception of the ones with irrelevant labels as flu-unrelated tweets.

#### Application Dataset

For validation purposes, we prepared an application dataset by collecting a set of Twitter posts for the first 20 weeks of the year 2018 within the boundary box of the state of Connecticut as a location filter using its associated longitude and latitude. The data were collected from Twitter SNS using a crawler that works with the Twitter API to stream tweets. The crawler is designed to filter the tweets based on keywords that are directly related to flu and verified by health care professionals. The list contains 11 flu-related keywords: fever, headache, sick, respiratory virus, ache, stuffy nose, dehydration, flu, influenza, contagious, and cough. Due to some technical issues, we were able to collect few twitter documents for the 10th week. Therefore, we did not include the period of the 10th week in our experiments. The total number of tweets over the 19 weeks was 8,440,670.

#### Centers for Disease Control and Prevention Influenza-Like Illness Network Data

ILI weekly rate produced by the CDC ILINet was used as a gold standard for comparison. The official ILI rates consider outpatients with symptoms of influenza who have visited any location of ILINet-participated health care providers around the United States. The data were obtained from the official CDC website [26].

#### Data of Hospital Emergency Department Syndromic Surveillance System

These data consist of the number of patients who have visited any location of the emergency departments (EDs) of the hospitals in Connecticut. Data of Hospital Emergency Department Syndromic Surveillance (HEDSS) generates daily reports about the daily patient visits based on the information received from the EDs. The generated reports include a percentage of patient visits for influenza [27]. These data are used to train the linear regression model for the final flu rate prediction for the state of Connecticut.

### Preprocessing

**Figure 2:**
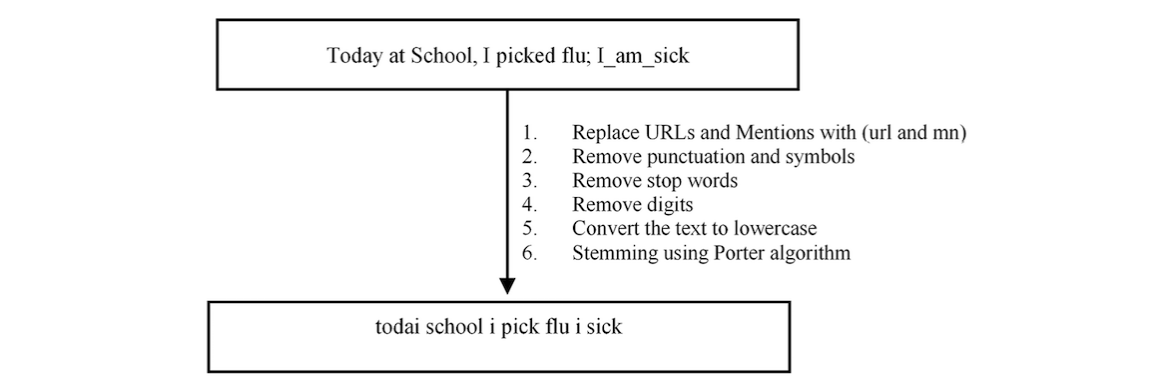
Text preprocessing.

During data preprocessing, stop-words, punctuations, and symbols were removed before the training and testing processes using the NLP toolkit (NLTK) [28]. Stop words such as “the” or “are” are very frequent and may lead to inaccurate classification results if used as features. The preprocessing also includes stemming that is used to reduce words to their roots. There are many stemming algorithms available for use. For this study, the stemming algorithm employed was Porter stemming. It is one of the most commonly used stemming algorithms. It is a rule-based algorithm with 5 steps that is designed based on the idea that English suffixes are made of smaller and simpler ones. A suffix is removed if a rule in the 5 steps passes the conditions and is then accepted [29]. Figure 2 shows the overall preprocessing steps.

URLs, hashtags, and mentions (MN) in the tweets were kept in the corpus. They can be used as features for classification. URLs were replaced with the keyword (url), and MN were replaced with the keyword (mn) to be used as one feature for classification.

### Feature Extraction

A maximum classification accuracy can be achieved by selecting the best set of features. Therefore, feature selection is a crucial process in any classification problem. In text classification, the set of features is a subset of words (*n*-gram) that can be used to distinguish between different classes. The selected words should provide useful information to be used for classification purposes. Thus, it is important to consider different techniques to convert the text in a way that can be processed to gain the required information. In this work, we considered additional features to enhance the classification accuracy. The additional features are sentiment based features, stylometric features, and flu-related keyword features (Algorithm 1 in Multimedia Appendix 1).

#### Textual Features

The default features in text classification are the terms and words that make up the document/text. Text classifiers are trained and tested using *n*-gram features, as basic features, by breaking down the documents/texts into single words (uni-grams), terms composed of 2 words (bi-grams), and terms composed of 3 words (tri-grams) and/or more. A basic technique in text classification is to count *n*-gram features including the uninformative ones that may yield inaccurate results. Therefore, it is important to use smarter techniques. One of these techniques is the word/term weighting technique, which weighs the count for every word/term in the text. There are different techniques of word weighting, which include Boolean weighting, term frequency weighting (TF), inverse document frequency weighting (IDF), and TF-IDF. Among the 4 types of word weighting techniques, only the IDF and TF-IDF techniques consider the importance of a word/term in the entire corpus instead of the importance of the word/term in only a document. It has been shown in [22] that TF-IDF is more accurate than IDF. Therefore, in this study, we used TF-IDF to weigh the *n*-gram features for the conventional machine learning classifiers.

**Figure 3:**
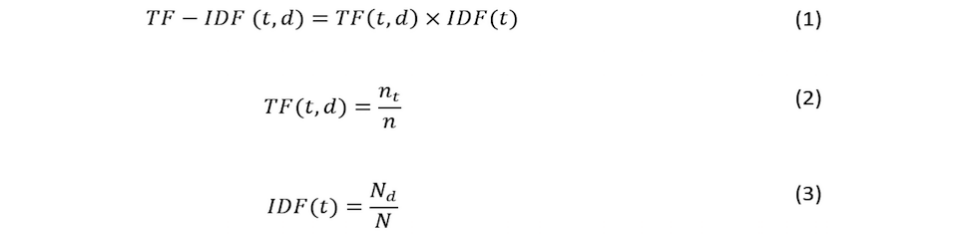
Term frequency-inverse document frequency calculations. TF: term frequency; IDF: inverse document frequency.

TF-IDF value is obtained by multiplying the TF value by the value of IDF (Equation 1 in Figure 3). *TF* is the ratio between the term *t* with frequency *n*_t_ in a given document *d* and the total numbers of terms *n* in the document *d* (Equation 2 in Figure 3). *IDF* is the inverse of the number of documents that has the term *t* at least once. It is calculated using Equation 3 in Figure 3, which is the ratio between the frequency *N*_d_ of the documents *d* that have term *t*, and the total number *N* of documents *d* in the analyzed corpus.

For the FT classifier, the representations of textual features of a document are averaged and weighted to be fed to the classifier. For word ordering, FT uses only partial information about the order by using bag of *n*-grams instead of BoW with the full information of the word ordering [30].

#### Stylometric Features

Stylometric features of twitter posts include retweets, MN, and URL links. These features were kept in the corpus to be used for classification. URL links and MN to others were preprocessed by replacing them to url and mn keywords.

#### Topic-Related Keywords-Based Features

 It is common to use seed words in text classification. For example, in sentiment analysis, a list of words, including nice and good, is used for positive sentiment and another list of words, including bad and poor, can be used for negative sentiment. In this study, a set of flu-related keywords/terms were used as a set of features for flu-related tweets. The list includes some important influenza-related keywords, symptoms, and treatments. The list of the keywords is kept in an array, and then each tweet is compared against these keywords to keep track of their occurrences.

#### Sentiment-Based Features

Sentiment analysis is the process of extracting the sentiment of a text using contextual polarity. It is commonly used in classifying reviews of different products in the Internet such as the sentiment of movies. In this study, we used TextBlob library to assign a sentiment to each tweet [31]. TextBlob is a Python library that is used to analyze textual data. On the basis of the polarity score of a tweet, a sentiment value is assigned to the text: positive or negative.

### Classification Model Building: Training and Testing

For the sake of accuracy and efficiency, various classifiers are evaluated, including FT and 6 conventional machine learning algorithms [32,33].

#### FastText

FT is a text classifier. It has been shown that FT produces accurate classification results that are comparable with the results produced by deep neural network classifiers. In addition, it has been shown that the processes of FT training and classification are very fast using a standard computer with a multicore processor. An FT model can be trained using billions of words in just a few minutes, and it can classify about 500,000 sentences in less than a minute [30].

FT utilizes several techniques to enhance the efficiency. It is a linear-based model, scaled to very large data and large output space using a rank constraint and a fast loss approximation. It uses a hierarchal softmax function for a faster search. In addition, only partial information about the word order is used for prediction. Furthermore, it uses the technique of hashing for textual feature mapping [30].

#### Conventional Machine Learning Classifiers

For training and testing, several supervised classification methods were evaluated to determine one with better classification accuracy [33]. The evaluated conventional classifiers include Random Forest, Naïve Bayes, SVM, C 4.5 Decision Tree, K-nearest neighbors (KNN), and AdaBoost. The preprocessed labeled dataset was used to train and test the model of different classifiers using 10-fold cross validation as the experimental setting. The 10-fold cross validation is a method to validate the studied/built model by iterating through the labeled data 10 times with different subsets of training and testing for each iteration.

### Mapping

For weekly rates, a MapReduce (MR) approach was used to process the large dataset of tweets. MR consists of 2 main functions: Map and Reduce. The Map function takes an input as a pair (week number and post), groups all the posts associated with the same week number, and generates intermediate pairs to be passed to the Reduce function. The Reduce function merges all the pairs with the same week number after processing the associated values such as counting or summing them up [34].

### Weekly Flu Rate Estimation Based on Regression

To predict the influenza rate at a certain week, we used a regression-based estimator. The proposed flu rate estimator has been evaluated using different regression models. In addition to the linear regression model, 3 different regression techniques were evaluated to determine the one with better estimation accuracy.

A regression model should be trained (fitted) using available data of flu rates, such as the data obtained from FluNearYou [35]–a Web application that uses weekly surveys to collect health status of individuals or the data of flu emergency visits obtained from HEDSS. For this study, we used the data of HEDSS for regression model training, where the average ILI rates of previous years and rates of flu-related tweets obtained from the classification results are passed to the regression model as predictors. The regression model was then tested and validated using CDC ILINet data.

#### Linear Regression Model

Linear regression is used when the dependent variable (response) is continuous and the independent variables (predictors) are either continuous or discrete, and the relationship between the dependent and independent variable(s) is linear. The linear regression indicates that the rate in the change of the mean of the response value is constant with respect to the value of the predictor(s). Therefore, the relationship is represented by an equation of a line [36].

**Figure 4:**
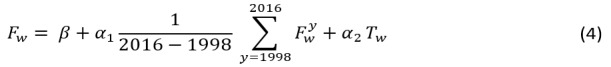
Proposed linear regression model.

Using the proposed predictors that include a combination of the rate of flu tweets and the average ILI rate of the same week number of past years (from 1998 to 2016), our proposed linear regression model has the following form (Figure 4), where indicates the flu rate at week *w*, *β* is the intercept which is the mean value of when all predictors are (0), values represent the regression coefficients, is the actual rate of flu incidents in week *w* of year *y*, and is the rate of flu tweets in week *w*.

#### Other Regression Models

In addition to our proposed linear regression model, 3 different regression techniques were evaluated to determine the technique with better estimation accuracy. The evaluated techniques are polynomial regression, logistic regression, and support vector regression. The measure of Pearson correlation (*r*) is used to find the most accurate model to be used for the final weekly flu rate estimation.

## Results

### Classification Results

**Figure 5:**
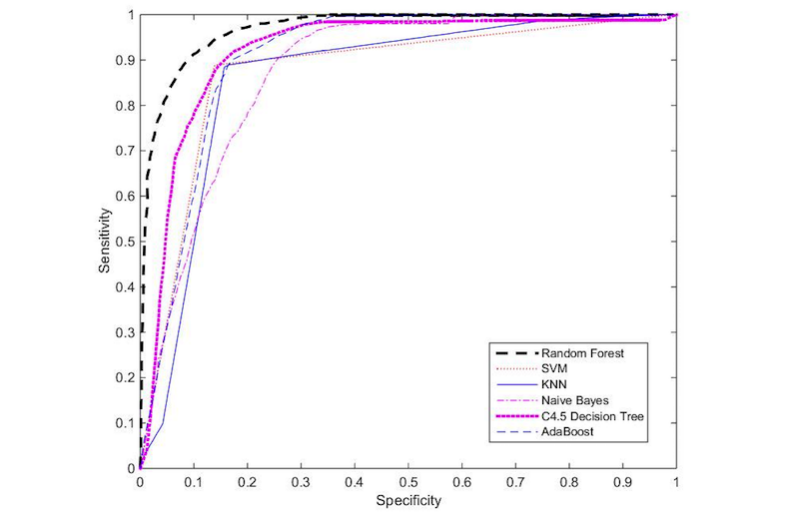
Performance comparison using receiver operating characteristic. SVM: support vector machine; KNN: K-nearest neighbors.

The results show that the proposed model improves the performance of flu post classifications using a combination of the additional features. The performance results of the used classifiers are shown in Table 1 using the precision, recall and *F*-measure metrics. The Random Forest method achieved the highest accuracy results, with an *F*-measure of 90.1*%.* In addition, we used the receiver operating characteristic (ROC) metric to evaluate the used classifiers. ROC is a curve with points that represent the pair of TP rate (sensitivity) and false positive rate (specificity). A perfect curve is the one that passes through the upper left corner representing 100*%* sensitivity and 100*%* specificity. Thus, the closer the curve is to that corner, the better the accuracy is [37]. As shown in Figure 5, Random Forest appears to be the best classifier. The high accuracy results demonstrate the efficiency and effectiveness of the extracted features.

**Figure 6:**
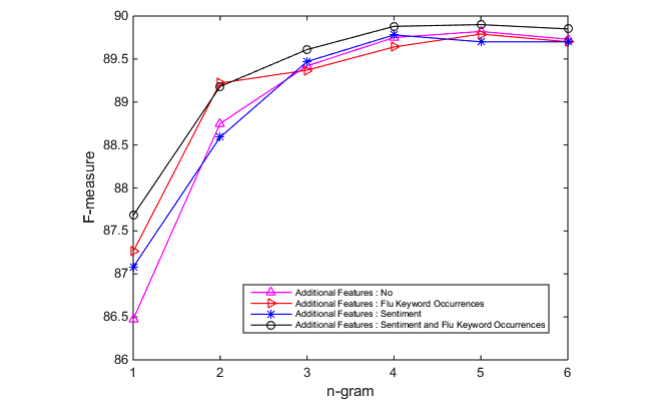
FastText performance using different sets of features.

Moreover, the performance results of FT with different sets of features, is presented in Figure 6. The overall accuracy using the *F*-measure metric ranges between 86.47*%* and 89.9%. This demonstrates the efficiency of the FT classifier. The highest classification accuracy is achieved by using the 5*-* gram features, together with all the proposed additional features (*F*-measure=89.9*%*) in only 21.53 seconds for training and testing using 10-fold cross validation on a standard computer (2.6 GHz Intel Core *i7* processor, and 16 GB RAM). It has been shown that FT can produce, in a short time, accurate results that are comparable with the results produced by the state-of-the-art deep neural network classifiers [30]. The high accuracy, together with the efficiency of FT make it an optimal classifier for flu disease surveillance models/systems with very large data. Therefore, FT will be used for our further analysis.

**Table 1:** Performance of classifiers.

Classifier name	Precision	Recall	*F*-measure
C4.5 Decision Tree	0.876	0.85	0.873
Random Forest	0.905	0.902	0.901
Support vector machine	0.883	0.883	0.883
Naïve Bayes	0.846	0.826	0.824
AdaBoost	0.867	0.864	0.864
K-nearest neighbors	0.874	0.872	0.872
FastText	0.899	0.899	0.899

Many studies have used the available data from Twitter to build faster influenza surveillance systems [17]. All the reviewed studies use conventional machine learning methods to distinguish between flu-relevant and flu-irrelevant posts for further analysis. A summary of the performance results of previous works, which include tweet classification for Twitter-based flu surveillance systems, is shown in Table 2. The metrics are reported as percentages. The evaluation of flu tweet classification using the *F*-measure shows that the proposed framework using FT, together with the extracted features, achieved high accuracy with *F*-measure value of 89.9%.

**Table 2:** Summary of the reviewed flu posts classifiers (flu-relevant/flu-irrelevant).

Study	Classifier name	Precision	Recall	*F*-measure	Note
Broniatowski et al [18], Lamb et al [19]	SVM^a^ and Logistic Regression	67	87	75.62	Multilevel classification
Santos and Matos [21]	Naïve Bayes	N/A^b^	N/A	83	—^c^
Aramaki et al [20]	SVM	N/A	N/A	75.6	—
Cui et al [22]	SVM	87.49	92.28	89.68	—
Byrd et al [23]	Naïve Bayes	N/A	N/A	N/A	70% accuracy
Proposed framework	Random Forest	90.5	90.2	90.1	—
Proposed framework	FastText	89.9	89.9	89.9	—

^a^SVM: support vector machine.

^b^N/A: not applicable.

^c^Not available.

### Weekly Flu Rate Estimation Results

The framework was evaluated by applying the trained FT model on the application data, which includes over 8,400,000 tweets, for classification. Then, the classification results together with the historical CDC data were passed on to the proposed regression-based estimator as predictors to obtain weekly flu-rates. The results of the flu estimator show a highly correlated output to the gold standard data (CDC). The estimator was evaluated using several regression models. Every model was fitted using the data of flu emergency visits obtained from HEDSS. Then, it was tested on CDC ILINet data from January 1, 2018 to May 19, 2018.

The performance results of the proposed flu rate estimator based on different regression models are shown in Table 3. The table presents the accuracy results using the Pearson correlation measure *r*.

**Figure 7:**
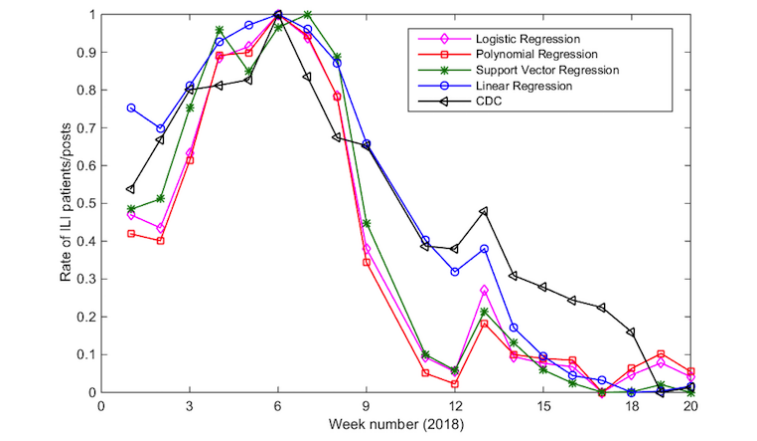
Correlation between the proposed framework and CDC influenza-like illness rate using different regression models. CDC: Centers for Disease Control and Prevention; ILI: influenza like illness.

The linear regression-based estimator achieved the highest accuracy results, with a Pearson correlation of 96.2%. Figure 7 also shows that linear regression is the most correlated model with the ground truth (CDC). It shows the normalized rate of ILI patients obtained from the CDC and the normalized rate of ILI Twitter posts obtained from the output of our proposed solution during the period of January through May of 2018 for the state of Connecticut. The rate values of the proposed framework and ILINet are normalized to a common scale for comparison.

**Table 3:** Performance of flu rate estimator using different regression models.

Regression model	*r* value
Polynomial regression	0.895
Logistic regression	0.917
Support vector regression	0.930
Linear regression	0.962

## Discussion

### FastText Versus Conventional Machine Learning Classifiers

To build a classification model with better accuracy and efficiency, FT and several supervised classification methods using the proposed additional features were evaluated. In addition to FT, the evaluated classifiers are Random Forest, Naïve Bayes, SVM, C4.5 decision tree, KNN, and AdaBoost. The preprocessed labeled dataset was used to train and test models of the different classifiers with the TF-IDF–based *n*-gram features and the proposed additional ones, which are presented in the Feature Extraction Section.

### Computational Complexity

The experiments show that FT produces accurate classification results in only 21.53 seconds for training and testing using 10-fold cross validation on a standard computer (2.6 GHz Intel Core *i7* processor, and 16 GB RAM). FT is an efficient linear-based model. It uses a hierarchal softmax function that reduces the computational complexity to become logarithmic *O* (log*n*), leading to faster classification training and testing [30]. For word ordering, only partial information about the order is used by using a bag of *n*-grams instead of a BoW with the full information of the word ordering. For more efficiency, the bag of *n*-grams are mapped using hashing techniques [30]. On the other hand, the experiments show that Random Forest, which is the most accurate conventional classifier in our experiment with F-measure value of 90.1, requires a longer time (39 min and 26 seconds) for training and testing using the experimental settings. The worst time complexity of Random Forest is quadratic for training *O* (*n*^2^log*n*) and linear for prediction *O* (*n*) [38]. This, together with the experimental results, demonstrates the efficiency and the accuracy of FT classifier. FT is an optimal classifier to detect new outbreaks with new signs and symptoms published in posts of SNSs. Therefore, FT has been adopted for further analysis in our proposed framework.

### FastText as a Flu Post Classification Module

For a better FT model, we evaluated 28 different feature settings using FT, with the parameter values of learning rate of 0.8 and epoch of 8, to determine the best feature set. Initially, the model was trained and tested using 1 setting of *n*-gram features (*n=* 1*-* 6), which are tokens of (*n*) words including the stylometric features. Then, different settings of the additional features are combined with the tweet text for training and testing using *n*-grams (*n=* 1-6). The settings include a combination of text and sentiment features, a combination of the text and keyword occurrence features, and a combination of all additional features (text + sentiment + hasKeyword):

__label__<related/unrelated>TEXT _sent_<neg/pos> _hasKeywrd_<yes/no>

With a standard computer (2.6 GHz Intel Core *i7* processor, and 16 GB RAM), the preprocessed labeled dataset was used to train and test the models using 10-fold cross validation as well.

### Linear Regression as a Weekly Flu Rate Estimation Module

**Figure 8:**
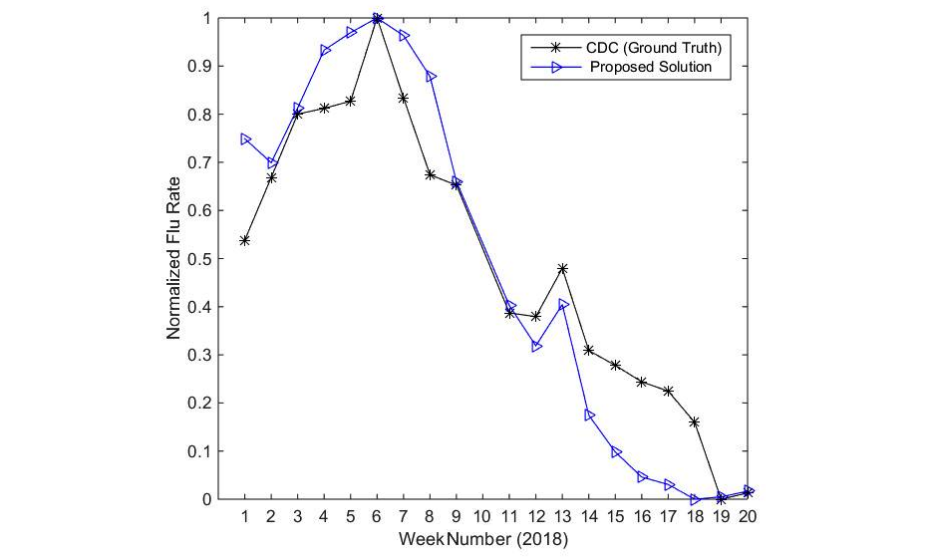
Correlation between the proposed framework and CDC influenza-like illness rate. CDC: Centers for Disease Control and Prevention.

In addition to the efficiency of linear regression, the experimental results, as shown in Figure 8, demonstrate the model accuracy and confirm the linear relationship between the rates of weekly flu (dependent variable) and flu-related tweets (independent variable). Therefore, the linear regression model is used for the weekly flu rate estimation module.

### Statistical Power Analysis

**Figure 9:**
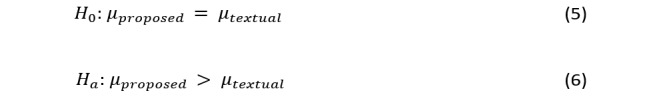
The 2 opposing hypotheses.

Power analysis has been performed to justify and ensure the appropriateness of the number of instances that are used for this study. Experimental results show that the accuracy of flu tweet classification using FT with the proposed additional features outperform FT with only textual features. Therefore, power analysis is also used to prove this hypothesis, which is stated as an alternative hypothesis *H*_a_, whereas the null hypothesis *H*_0_ is the hypothesis where there is no change in the accuracy using proposed features with respect to only textual features. With the power analysis, a statistical test rejects the null hypothesis when it is false. With this, one can conclude that there is a difference between the accuracies (better accuracy) using additional features and can confirm our alternative hypothesis *H*_a_. If the null hypothesis is not rejected, then the alternative hypothesis should be rejected. The opposing hypotheses for our work can be stated as shown in Figure 9, where *µ*_proposed_ is the accuracy average of FT using the proposed additional features, and *µ*_textual_ is the accuracy average of FT using only textual features for flu tweet classification.

To determine the required sample size *n*, 4 parameters/factors must be known or estimated, which are as follows:

- α: significance level (1% or 5%)
- p: desired power of the test (80%)
- σ: population SD
- d: effect size (the difference between the 2 groups)

The values of the first 2 parameters are generally fixed. The parameter of significance level α is usually set to either .05 or .01 and is the probability of rejecting the null hypothesis when it is true. The power parameter *p* is the probability that the effect will be detected and is usually set to either 0.8 or 0.9. On the other hand, the last 2 parameters are problem dependent. For our analysis, the last 2 parameters are estimated based on our previous experiments. Thus, the values of all the 4 required parameters are stated below:

- α=5%
- p=80%
- σ=0.27
- d=0.012

**Figure 10:**
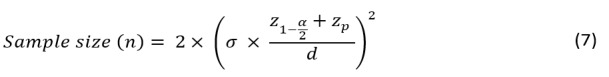
Sample size formulation.

Using these parameters together with the *z*-test model to obtain *z*-scores, the sample size *n* can be computed by using Equation 7 (Figure 10).

**Figure 11:**
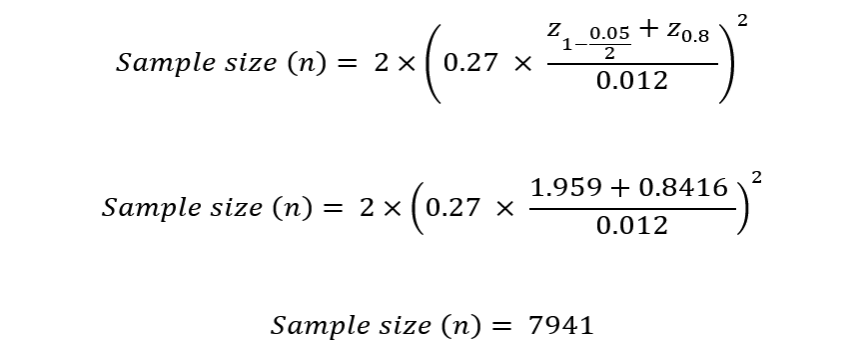
Sample size computation results.

Given the estimated values of the required parameters, we will obtain the computations and values shown in Figure 11.

**Figure 12:**
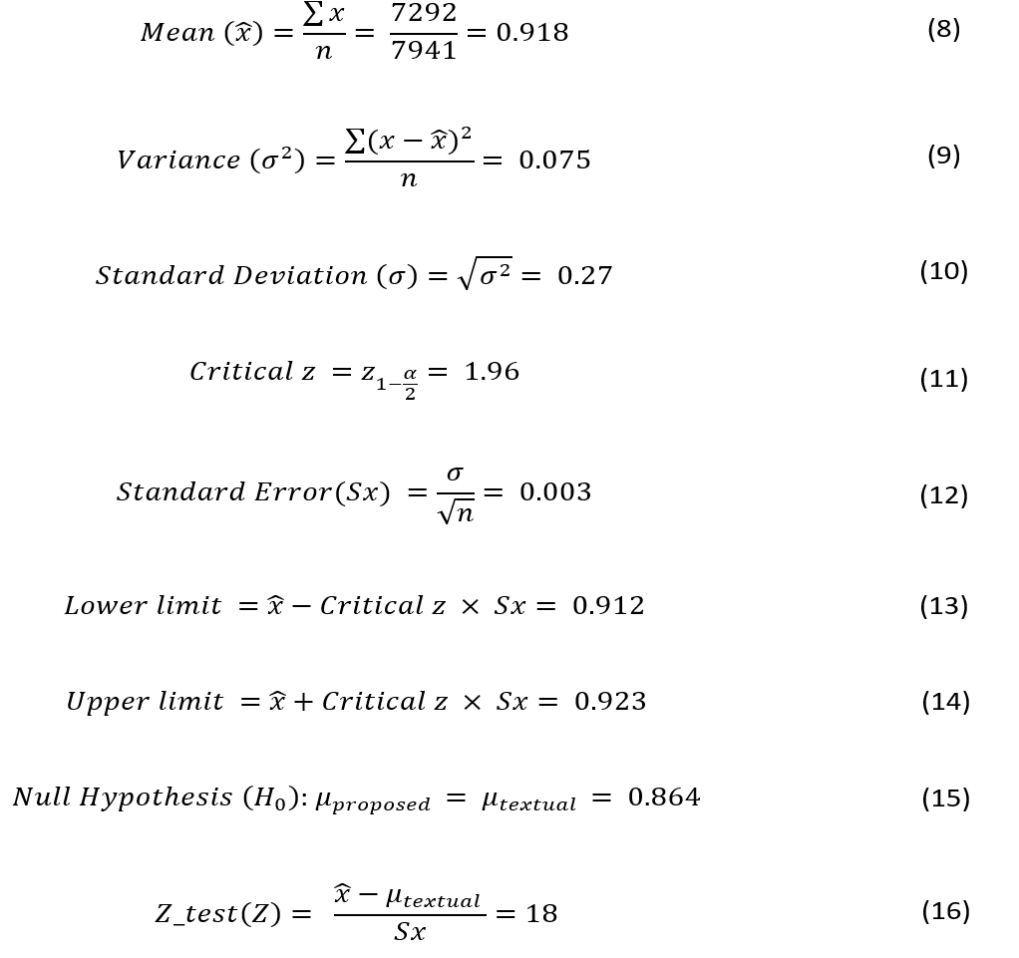
Z-test computation and analysis to determine whether to accept or reject the alternative hypothesis.

Using the obtained sample size *n* and the significance level *α*, the below parameters (in Figure 12) can be computed to apply the *z*-test and then make a decision on accepting or rejecting our alternative hypothesis.

Because the obtained value of the *z*-test (18) is higher than the critical value (18>1.96), the observed difference is significant and shows that the additional features enhance the accuracy of FT to classify flu tweets. In other words, results of the *z*-test show that the null hypothesis (H_0_) should be rejected, and the sample set of 7941 tweets is sufficient to prove that FT with the proposed additional features is more accurate than FT with only textual features for flu tweet classification. Our experiments included over 10,000 tweets, which is more than enough to prove the hypothesis claims.

### Performance Metrics

**Figure 13:**
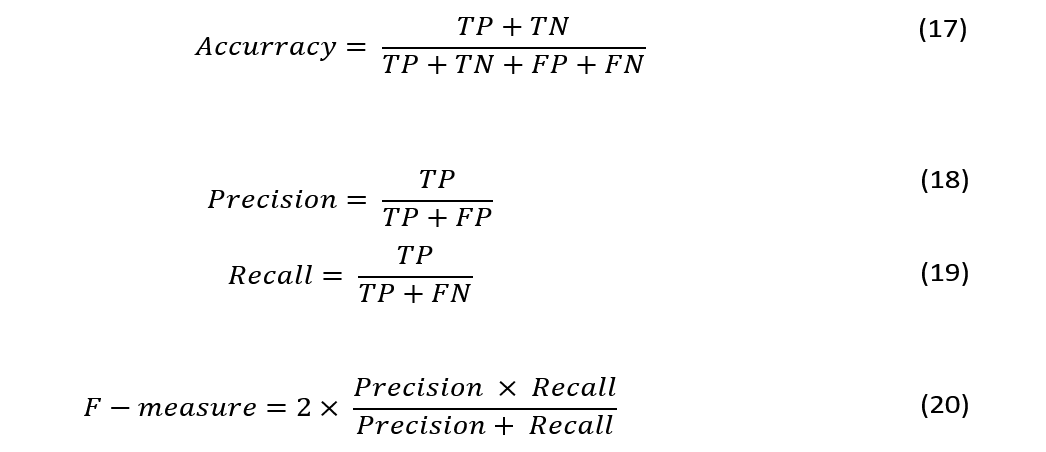
Performance metrics used to evaluate the proposed work. FN: false negative; FP: false positive; TP: true positive.

In this section, we present the used performance metrics. The performance of the classifiers are evaluated using different metrics presented in Figure 13, which are as follows: accuracy (Equation 17), precision (Equation 18), recall (Equation 19), and *F*-measure (Equation 20). These metrics are used to provide a better overview of the model performance. The accuracy measure by itself is not a perfect measure if the dataset is not balanced. Precision and recall are better measures in the case of imbalanced datasets. The selected metrics can be computed using true positive (*TP*), true negative (*TN*), false positive (*FP*), and false negative (*FN*) measures, where *TP* refers to the rate of correctly classified instances as positive, *TN* refers to the rate of correctly classified instances as negative, *FP* refers to the rate of incorrectly classified instances as positive, and *FN* refers to the rate of incorrectly classified instances as negative. In this work, we mainly use *F*-measure as a performance metric for evaluation and comparison. *F*-measure is a weighted average of 2 different performance metrics: precision and recall. Its value ranges between 0 (worst) and 1 (best).

**Figure 14:**
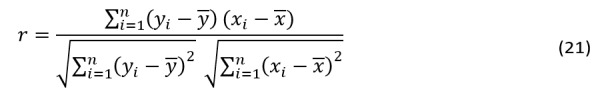
Pearson correlation value computation.

In addition, the performance of flu rate estimation is evaluated using Pearson correlation. It is a metric that evaluates the correlation between 2 datasets using the symbol *r* that ranges between (1) and (*−* 1): the value of *r=* 1 when both datasets exactly match and the value of *r=* 0 when there is no correlation between the 2 datasets. An available ground truth is usually used to evaluate the quality of the results of the proposed methods and frameworks. For this study, we used the recent CDC weekly reports as the ground truth to be compared with the proposed solution. Let be the observed value of the ground truth (CDC ILINet data), *x*_i_ be the predicted weekly flu rate value, and *y* ¯ and *x* ¯ be the average values of *y*_i_ and *x*_i_, respectively. Using these notations, the Pearson correlation value *r* is defined as shown in Equation 21, illustrated in Figure 14 [39].

As shown in Table 4 and depicted in Figure 8, the results show a strong correlation (96.29*%* Pearson correlation) between the output of the proposed framework and the CDC reports. This correlation percentage shows that our proposed solution provides accurate results on par with the best results in our survey, while being more efficient (faster). In addition, we believe that this is the first work that uses Twitter postings for flu trend predictions in the state of Connecticut with strong correlated results. To the best of our knowledge, this is also the first work that shows a Twitter-based solution for flu prediction using recent data that were collected in the year of 2018.

**Table 4:** Summary of the reviewed studies with reported Pearson correlation.

Study	Time frame	Location	*r* value
Broniatowski et al [18]	September 2012-May 2013	United States	0.93
Aramaki et al [20]	November 2008-June 2010	Japan	0.89
Santos and Matos [21]	March 2010-February 2012	Portugal	0.89
Lamb et al [19]	May 2009-October 2010	United States	0.9897
Cui et al [22]	September 2013-December 2013	China	—^a^
Byrd et al [23]	October 2015-November 2015	Ottawa	—
Proposed framework	January 2018-May 2018	Connecticut, United States	0.9629

^a^Not applicable.

### Conclusions

For disease surveillance models, gathering related information about diseases and then issuing necessary reports at an early stage is crucial for outbreak prevention. Data of microblogging sites, such as Twitter, have become popular enough to be used as triggers for different event prediction such as disease outbreaks. Recently, many studies have used these data to build effective epidemic prediction models such as flu outbreak prediction. It has been observed in the literature that most of the models use conventional machine learning methods to filter and distinguish between the flu-relevant and irrelevant posts for further analysis. In this study, we introduced a framework based on FT, a state-of-the-art text classifier that uses the features of sentiment analysis and flu keyword occurrences for faster classification. Thereafter, a combination of the classified Twitter documents and historical CDC data was passed to a linear regression–based module for weekly flu rate predictions. The results demonstrated the efficiency and the accuracy of the proposed framework. The final predicted flu trend using Twitter documents showed a strong Pearson correlation of 96.29 *%* with the ground truth data of CDC for the first few months of 2018.

## Appendix

Multimedia Appendix 1 Additional feature extraction algorithm.

## Abbreviations

CDC: Centers for Disease Control and Prevention
ED: emergency departments
FN: false negative
FP: false positive
FT: FastText
HEDSS: Data of Hospital Emergency Department Syndromic Surveillance
IDF: inverse document frequency
ILINet: Influenza Like Illness Surveillance Network
KNN: K-nearest neighbors
MI: mutual information
MN: mentions
MR: MapReduce
NLP: natural language processing
NLTK: natural language processing toolkit
ROC: receiver operating characteristic
SNSs: social networking sites
SVM: support vector machine
TF: term frequency
TF-IDF: term frequency-inverse document frequency
TN: true negative
TP: true positive
