# Supplementary material for: Preliminary Flu Outbreak Prediction Using Twitter Posts Classification and Linear Regression With Historical Centers for Disease Control and Prevention Reports: Prediction Framework Study
Source: JMIR Public Health Surveill. 2019 Jun 23;5(2):e12383. doi: 10.2196/12383 (PMC6615001; doi:10.2196/12383)
Supplement: Multimedia Appendix 1 [file publichealth_v5i2e12383_app1.pdf]

Algorithm 1 Additional Feature Extraction

```
twt_txt ← tweet_document.text
Txtlen ← length of twt_txt

Feature Set1:                          %Preprocessing and stylometric Feature Extraction
 if URL  in twt_txt  then
     twt_txt ← replace URL with a keyword url
 if Mention  in twt_txt  then
     twt_txt ← replace Mention with a keyword mn
 Token ← tokenize(twt_txt)
 for (i = 0; i < txtlen ; i = i+1)  do
     if (token(i)  is ineffective char) OR ( token(i)  in Stop_Word_lst) then
         remove token(i)
     stem ( lower (token (i))
 twt_txt ← token

Feature Set2 :                         %Sentiment Feature Extraction
 sent_ f t ← 0
 Polarity_score ← find_polarity(twt_txt)
 if Polarity_score > 0  then
     sent _ f t = 1
 Else
     sent _ f t = 0

Feature Set3 :                         %Keyword Occurrences Feature Extraction
 hsKwrd_ f t ← 0
 kwrd_lst_len ← length of keyword_lst
 for (i = 0; i < kwrd_lst_len ; i = i+1)  do
     if keyword_lst(i)  in twt_txt) then
         hsKwrd _ f t = 1
 twt_txt_w_features ← concatenate(twt_txt, _sent_(sent_ft), _hsKwrd_(hsKwrd_ft) )
```
